# Novel Acrylic Bone Cement Containing Graphene Oxide: Synthesis and Characterization

**DOI:** 10.3390/polym18010131

**Published:** 2025-12-31

**Authors:** Luiz Fabiano Gomes Gularte, Guilherme Kurz Maron, Camila Perelló Ferrúa, Andressa da Silva Barboza, Tiago Fernandez Garcia, Geovanna Peter Correa, Cainá Corrêa do Amaral, Bruna Godinho Corrêa, Chiara das Dores do Nascimento, Everton Granemann Souza, Cesar Aguzzoli, Neftali Lenin Villarreal Carreño, Juliana Silva Ribeiro de Andrade, Rafael Guerra Lund, Fernanda Nedel

**Affiliations:** 1 Graduate Program in Health and Behavior, Catholic University of Pelotas, Pelotas 96015-560, Brazil; luizgularte@gmail.com (L.F.G.G.); camila_perello@hotmail.com (C.P.F.); tiagogarcia.fisio@gmail.com (T.F.G.); geovannapeter_@live.com (G.P.C.); caina.ca@hotmail.com (C.C.d.A.); bruna.godinho@sou.ucpel.edu.br (B.G.C.); 2 Graduate Program in Science and Materials Engineering, Technology Development Center, Federal University of Pelotas, Pelotas 96010-610, Brazilchiaradnascimento@gmail.com (C.d.D.d.N.); nlv.carreno@gmail.com (N.L.V.C.); 3 Graduate Program in Dentistry, Pelotas Dental School, Federal University of Pelotas, Pelotas 96015-560, Brazil; andressahb@hotmail.com (A.d.S.B.); sribeirooj@gmail.com (J.S.R.d.A.); 4 Graduate Program of Electronic and Computer Engineering, Catholic University of Pelotas, Pelotas 96015-560, Brazil; everton.granemann@ucpel.edu.br; 5 Exact Sciences and Engineering, University of Caxias do Sul, Caxias do Su 95070-560, Brazil; caguzzol@ucs.br

**Keywords:** polymethyl methacrylate, graphene oxide, bone cements, biocompatible materials, anti-bacterial agents

## Abstract

Polymethylmethacrylate (PMMA) bone cement is widely used in orthopedics, accounting for approximately 80% of knee joint replacements in the United States. While prosthesis designs and materials have evolved to improve performance and durability, PMMA cement has undergone minimal compositional changes. Carbon-based nanomaterials, particularly graphene oxide (GO), have attracted interest for their ability to enhance the mechanical and thermal properties of orthopedic cements. This study evaluated the effects of incorporating different GO concentrations into PMMA bone cement on its mechanical properties, cytocompatibility, and antibacterial activity. PMMA was modified with GO at 0.1, 0.25, and 0.5 weight percent (wt%) for mechanical and antibacterial tests, and at 1.0 wt% for cytocompatibility. Mechanical performance was assessed via four-point bending tests. Cytocompatibility was evaluated using mouse embryonic fibroblasts (NIH/3T3), and antibacterial activity was tested against *Staphylococcus aureus* using a modified direct contact assay. GO incorporation significantly increased Young’s modulus (0.1% and 0.25%, *p* = 0.009) and improved tensile strength (*p* = 0.0015) and flexural strength (*p* = 0.025) at 0.1%. Cytocompatibility remained comparable to the control (*p* = 0.873). Antibacterial activity was concentration dependent, with 0.25% and 0.5% GO maintaining significant bacterial inhibition up to 48 h, whereas 0.1% showed no sustained effect. Overall, 0.25 wt% GO provided the most suitable balance between mechanical integrity and antibacterial performance, indicating that PMMA–GO bone cements with this composition can combine enhanced mechanical properties with relevant antibacterial activity without compromising biocompatibility, and are therefore promising candidates for orthopedic applications.

## 1. Introduction

Joint replacement surgeries in the lower limbs are considered the most successful orthopedic procedure, with several reports of more than 90% implant survival in 15 to 20 years [1,2,3]. However, about 1–2% of patients with joint replacement procedures develop infection and osteomyelitis as a complication [4], generating extremely high costs per patient [5,6], often resulting in loss of implant or loosening.

The socioeconomic impact of joint replacement procedures is highly significant, deeply affecting the quality of individuals’ lives and causing an economic burden on the health system. Over time, designs of the prostheses and the materials that make them up have evolved to achieve improved performance and increased durability. However, although a large part of the prostheses is fixed to the bone through acrylic cement, the latter has not had major changes in its composition over time [6].

Polymethylmethacrylate (PMMA) has been a cornerstone material in orthopedic surgery for decades, primarily used as bone cement to anchor prosthetic implants to bone. Its widespread adoption is attributed to its excellent biocompatibility, ease of handling, and rapid in situ polymerization [7]. Nevertheless, despite its long-standing clinical success, PMMA presents inherent limitations, including suboptimal mechanical strength (particularly in tension and fatigue), exothermic polymerization capable of causing thermal necrosis, lack of bioactivity, and susceptibility to bacterial colonization, which can lead to prosthetic joint infection (PJI) [8,9].

One of the main reasons for this is the fact that any material added to the PMMA formula causes a variable change over its main properties, which translates into a worsening of its main characteristics like tensile strength, bending strength, bending modulus and Young’s modulus [7]. Recent research has focused on nanoscale reinforcements to overcome these drawbacks without significantly altering the cement’s handling properties [7,8].

Carbon-based nanomaterials are a particular category of nanometer-sized material. The use of these nanomaterials in low concentrations has been widely studied for a wide variety of applications due to their ability to improve the mechanical and thermal properties of orthopedic cement [7], especially in the form of graphene. Graphene is a sp2 hybridized carbon allotrope, which comprises a single monocrystalline atomic layer of six-atom rings in a two-dimensional honeycomb structure network, where the carbon atoms remain connected by van der Waals forces to a distance of about 0.335 nm [8], resulting in a large surface area on both sides of the planar axis [9,10].

Graphene properties are those of semi-metals and are stable under ambient conditions. This contradicts the general belief that a 2D material could not exist and be thermodynamically stable [8]. Its unique structure induces free π electrons, which facilitates its high electrical conductivity (on the order of 10^4^ S·cm^−1^, a value significantly higher than that of polymeric matrices). In addition, it has incomparable mechanical resistance, with a very high elastic modulus (1000 GPa), optical transmittance (97.7%), a large specific surface area (2630 m^2^/g), and excellent thermal conductivity (≈5000 W mK^−1^) [11]. Due to these special characteristics, together with flexibility in chemical and biological functionalization, adaptability to flat or irregular surfaces, and simplicity in mass production, intensive research on graphene and its derivatives for biological studies has been carried out, and the materials have demonstrated potential utility in many biomedical applications [12,13,14,15,16].

Graphene oxide (GO) is a sheet of graphene with a range of functional groups at its edges and in its basal plane, such as carboxyl, hydroxyl, and epoxide groups that allow GO to interact with proteins or other molecules through covalent and non-covalent bonds [17]. The layered GO is more hydrophilic than graphene because its polar functional groups that contain oxygen on the nanolayer surface establish hydrogen bonds with water molecules, offering reasonable colloidal dispersibility under suitable pH conditions [18]. Within this perspective, extensive research has been done showing that GO has osteogenic and bactericidal properties [19,20,21,22], which could prevent bacterial adhesion and stimulate host bone growth at the bone-cement interface, increasing the fixation of the prosthesis and presenting a deep positive impact on arthroplasties. While previous studies have explored GO-PMMA composites, most have focused on a single property or a limited range of GO concentrations [7,11,13,19,22]. The novelty of this work lies in the systematic and concurrent evaluation of mechanical performance, cytocompatibility, and concentration-dependent antibacterial activity across multiple GO loadings (0.1, 0.25, 0.5 wt%). This integrated approach provides a more comprehensive understanding of the structure-property relationships in PMMA-GO composites and clarifies the trade-offs between mechanical reinforcement and antibacterial efficacy. Our hypothesis is that the incorporation of an optimized GO concentration will simultaneously enhance the mechanical properties and introduce concentration-dependent antibacterial activity without compromising the cytocompatibility of the bone cement. Thus, our study aimed to show that the association of GO with PMMA at different concentrations may lead to an improvement in the physical characteristics of the cement and bacterial inhibition.

## 2. Materials and Methods

Sulfuric acid (H_2_SO_4_; Synth, São Paulo, SP, Brasil), graphite (98%; Synth, SP, Brazil), sodium nitrate (NaNO_3_; Synth, São Paulo, SP, Brazil), potassium permanganate (KMnO_4_; Synth, São Paulo, SP, Brazil), hydrogen peroxide (29%, H_2_O_2_; Synth, São Paulo, SP, Brazil), and radiopaque bone cement (Cimtech^®^, São Paulo, SP, Brazil), specifically the PMMA powder component (MW ~200,000 g/mol, purity > 99.5%) and methyl methacrylate liquid monomer (stabilized with hydroquinone), were purchased. Mouse fibroblast cells (NIH/3T3) were obtained from Rio de Janeiro Cell Bank (PABCAM; Federal University of Rio de Janeiro, Rio de Janeiro, RJ, Brazil). Dulbecco’s modified Eagle’s medium (DMEM; Gibco, Grand Island, NY, USA), fetal bovine serum (FBS; Gibco, Grand Island, NY, USA), 3-(4,5-dimethylthiazol-2-yl)-2,5-diphenyltetrazolium bromide (MTT; Sigma-Aldrich, St. Louis, MO, USA), and dimethyl sulfoxide (DMSO; Sigma-Aldrich, St. Louis, MO, USA), Tryptic Soy Agar (TSA KASVI; Belo Horizonte, MG, Brazil), Tryptic Soy Broth (TSB; KASVI; Belo Horizonte, MG, Brazil), were also purchased.

### 2.1. Preparation of Graphene Oxide (GO)

GO was synthesized based on the method previously described by Hummers and Offeman [23], via oxidation of graphite through a chemical treatment using sulfuric acid (H_2_SO_4_) and potassium permanganate (KMnO_4_). In this procedure, a solution containing 23 mL of H_2_SO_4_, 1 g of graphite, and 0.5 g of sodium nitrate (NaNO_3_) was kept under stirring in an ice bath at 0–5 °C for 30 min. Then, 3 g of KMnO_4_ was slowly added in small portions to control the temperature under 20 °C. The solution was kept stirring for 90 min, during which the mixture thickened and turned dark green. Subsequently, 46 mL of distilled water was added, causing a rapid temperature increase to approximately 98 °C for 15 min. Finally, 140 mL of distilled water and 10 mL of hydrogen peroxide H_2_O_2_ (29%) were added, and the solution was stirred for 15 min. The addition of H_2_O_2_ indicated the successful cessation of the oxidation, marked by a clear color change from dark brown to brilliant yellow. The final solution was centrifuged two times with a 10% hydrochloric solution and six times with ethanol, for 10 min at 3500 RPM. The final precipitate was dispersed in ethanol until its use.

### 2.2. Incorporation of GO into PMMA

The modification of PMMA was performed through the addition of different concentrations of GO (0, 0.1, 0.25, and 0.5% wt/wt of GO). In this procedure, a 5 mg/mL solution of GO in distilled water was dispersed using a bath ultrasound for 45 min. Then, the corresponding amount of PMMA powder was added, and the solution was maintained stirring for a further 60 min at room temperature. Finally, the resulting material was oven-dried at 50 °C for 24 h.

### 2.3. Polymerization of the Acrylic Bone Cement

The equipment, mixing surfaces, and materials were maintained at a temperature of 23 ± 1 °C and relative humidity of at least 40% for at least 2 h before testing. The polymerization was carried out according to the standards indicated by the manufacturer. Briefly, the powder was placed on a glass surface in a ratio of 2:1 to the liquid monomer, and subsequently, the liquid component was added to the powder. The components were mixed using an inert stainless-steel spatula (Teflon^®,^, The Chemours Company FC, LLC, Wilmington, DE, USA) for 1 min. The final groups were composed of polymerized acrylic bone cement (PMMA), with dimensions specified for each subsequent characterization, and entitled as PMMA, PMMA-GO 0.1%, PMMA-GO 0.25%, and PMMA-GO 0.5%, respectively, to the different concentrations of GO (0, 0.1, 0.25 and 0.5% wt/wt of GO) incorporated in the PMMA. It should be noted that a higher concentration (1 wt%) was intentionally not included in mechanical testing because preliminary trials and the literature evidence indicate that GO loadings ≥1% lead to agglomeration and structural defects in PMMA, compromising mechanical performance. This concentration was therefore reserved exclusively for the biological “worst-case scenario” required by ISO 10993-12.

### 2.4. GO Characterization

#### 2.4.1. X-Ray Diffraction (XRD)

X-Ray Diffraction (XRD 6000; Shimadzu, Japan) was performed to confirm the obtention of GO. Diffraction data were collected at a 2θ range from 10° to 70°, with a scanning rate of 0.5° per minute.

#### 2.4.2. Field-Emission Scanning Electron Microscopy (FESEM)

A field emission scanning electron microscope (FESEM, JSM-7500F, Jeol Ltd., Tokyo, Japan) was used to investigate the morphology of GO particles. Micrographs were obtained at different magnifications to assess both the general distribution of the particles (low magnification) and the specific surface morphology of the sheets (high magnification).

#### 2.4.3. Rutherford Backscattering Spectrometry (RBS)

The quantitative chemical composition of the samples was obtained by RBS using a 3 MV Tandem accelerator with a 2 MeV He^+^ beam incident perpendicular to the sample surface and a detector positioned at 165°, following previously established procedures [24,25]. RBS spectra were initially acquired as counts versus channel number using a multichannel analyzer. After energy calibration with standard samples, the channel axis was converted to an absolute energy scale (keV), as described in [26]. Medium- and high-Z elements were identified from the backscattered ion energy distribution. Light elements such as C and O were not reliably detected experimentally due to their low scattering cross-section, but were considered in the simulated profiles. The composition was estimated using a numerical fitting approach, using the SIMNRA software (version 7.03) [27], in which theoretical curves were iteratively adjusted to match the experimental data. The deviation between fitted and experimental spectra was below 5%.

### 2.5. Mechanical Tests

The bending tension, bending strength, bending modulus, and Young’s modulus of PMMA, PMMA-GO 0.1%, PMMA-GO 0.25%, and PMMA-GO 0.5% were determined by four-point bending tests, according to the standard ABNT NBR ISO 5833:2004, using a universal test machine (E3000, Instron, MA, USA). Five test specimens with dimensions of 75 mm in length, 10 mm in width, and 3.3 mm in thickness were prepared for each group of samples.

The four-point bending configuration followed ISO 5833:2004 and consisted of an outer support span (*L* = 60 mm) and an inner loading span (α = 20 mm), symmetrically positioned around the midline of the specimen. This configuration ensures a region of constant bending moment between the inner loading noses. A schematic illustrating all distances and loading points is provided in Supplementary Figure S1. The nominal stress (*σ*), or tensile were calculated according to the following equation:

(1) $$\sigma \;=\;\frac{F}{A}$$

where *F* is the force and *A* is the area of the specimen section. The values of bending strength (*B*) were calculated according to the equation below:

(2) $$B\;=\;\frac{3Fa}{bh^{2}}$$

where *F* is the force measured at the break, in Newtons, *a* is the distance between the inner and outer loading points (20 mm), *b* is the width and *h* is the thickness of the specimen. Values of bending modulus (*E*) were calculated using the following equation:

(3) $$E\;=\;\frac{\Delta Fa(3L^{2}-4a^{2})}{4fbh^{2}}$$

where *f* is the difference between the deflections under loads of 15 N and 50 N, *b* is the width of the specimen, *h* is the thickness of the sample, *L* is the distance between the external loading points (60 mm), *ΔF* is the load range (35 N) and *a* is the distance between the internal and external loading points (20 mm).

Although not a parameter specified in ISO 5833:2004, the bending-derived Young’s modulus ($E_{y}$) was additionally derived from the linear elastic region of the bending stress–strain curve and the values of *Ey* were calculated according to the following formula

(4) $$E_{y\;}=\frac{\sigma }{\varepsilon }\;$$

where *σ* is the stress exerted on the specimen and *ε* is the deformation suffered by the specimen.

### 2.6. Biological Assays

#### 2.6.1. Specimens Manufactured for Viability and Biofilm Inhibition Assays

Specimens were manufactured in a stainless-steel matrix with a 6 mm diameter and 2 mm thick (Odeme Dental Research, Lagoa Santa, MG, Brazil), thus creating specimens with 91.6 mm^2^ per milliliter of culture medium, according to the ISO 10993-12 standard. Every side of the specimens was sterilized under UV irradiation for 40 min. For the viability assay, a total of 24 specimens were manufactured, 12 per group (PMMA and PMMA-GO 1%) and 4 per period of exposure (1, 3, and 5 days). It is important to clarify that while mechanical tests utilized GO concentrations up to 0.5% to avoid structural defects, the viability assay employed a higher concentration (1% wt%) to strictly follow the ISO 10993-12 “worst-case scenario” recommendation. This ensures that if the highest concentration proves cytocompatible, the lower concentrations (0.1–0.5%) used in the mechanical and antibacterial groups are also biologically safe. The use of the highest concentration (1%) for the cytocompatibility assay was selected based on the ISO 10993-12 standard, which recommends a “worst-case scenario” evaluation to ensure maximal safety under stringent conditions. For the biofilm inhibition assay, a total of 36 specimens were manufactured, 12 per group (PMMA, PMMA-GO 0.1%, PMMA-GO 0.25%, and PMMA-GO 0.5%) and 3 per period of exposure (6, 24 and 48 h). This 1% group was not included in mechanical testing because such high loadings are known to induce agglomeration, porosity, and matrix defects, making the results non-representative of clinically acceptable PMMA formulations.

#### 2.6.2. Viability Assay

Cell viability assay was conducted to assess the effect of GO bone cement on NIH/3T3 mouse fibroblast cells. For this assay, a total of 24 specimens were manufactured, 12 per group (PMMA and PMMA-GO 1%) and 4 per period of exposure (1, 3, and 5 days). Each specimen was manufactured as described above and incubated with 1 mL of DMEM supplemented with 10% FBS, at 37 °C, under humidified 5% CO_2_ and 95% O_2_ for 1, 3, and 5 days. After these periods, specimens were removed and the eluate formed by the quadruplicate was combined and filtered (0.22 μm, Millex, Millipore, São Paulo, Brazil). The final eluates were then used to conduct the viability assay.

The viability of the NIH/3T3 cells was determined by measuring the reduction of soluble MTT to water-insoluble formazan [28,29]. Briefly, cells were seeded at a density of 2 × 10^4^ cells per well in a volume of 100 μL DMEM/10% FBS in 96-well plates and grown at 37 °C in a humidified atmosphere of 5% CO_2_ and 95% O_2_ for 24 h. The medium was discarded and 100 μL of the final eludate was added and incubated for an additional 24 h. After incubation, the medium was removed and 180 μL of DMEM and 20 μL of MTT (5 mg MTT/mL solution) were added to each well. Plates were incubated for an additional 4-hr period, and the medium was discarded. DMSO (200 μL) was added to each well, and the formazan was solubilized on a shaker for 5 min at 100× *g*. After homogenization, the absorbance of each well was determined on a microplate reader (MR-96A; Mindray Shenzhen, China) at 450 nm wavelength. All observations were validated by two independent experiments, and each experiment was performed in quadruplicate (*n* = 4). The negative control group consisted of DMEM/10% FBS without cells. The positive control group consisted of DMEM/10% FBS and NIH/3T3 cells (density of 2 × 10^4^ cells).

#### 2.6.3. Microbiological Assay

##### Modified Direct Contact Test

*Staphylococcus aureus ATCC 19095* was cultured overnight in TSB in aerobic conditions at 37 °C. After this period, this bacterial suspension was adjusted to 3 × 10^8^ CFU/mL. Each specimen (6 mm × 2 mm) was manufactured as described previously and disposed of in a 96-well plate (*n* = 4 samples per group, per time point). Then, 20 μL of the bacterial suspension was added to each well containing the samples and also to empty wells as a bacterial control. The 96-well plate containing the samples and the bacterial suspension was incubated for 6, 24, and 48 h at 37 °C under 100% relative humidity. After the proposed times, 180 μL of culture medium was added to each well and homogenized for 10 min. From this volume, 100 μL of the bacterial suspension from each well was transferred to an Eppendorf tube to perform decimal serial dilution in sterile saline (0.85% NaCl). The serial dilutions were plated in Petri dishes containing Tryptic Soy Agar (TSA). Each plate received four drops of 20 μL per dilution. The plates were then incubated at 37 °C for 24 h. Beyond the period of incubation, the Colony Forming Units (CFU/mL) were counted. The lower limit of detection for this assay was 102 CFU/mL. The antibacterial rate was calculated based on the reduction in CFU/mL compared to the PMMA control group at each time point.

### 2.7. Statistical Analysis

Tensile strength, bending strength, bending modulus, and Young’s modulus results were evaluated by statistical analysis using one-way analysis of variance (ANOVA). Biofilm inhibition and cell viability results were evaluated by two-way ANOVA. All analyses were performed at a significance of α = 0.05 followed by the Tukey test to evaluate the difference between groups. All groups were compared with each other for each analysis.

## 3. Results

**Figure 1:**
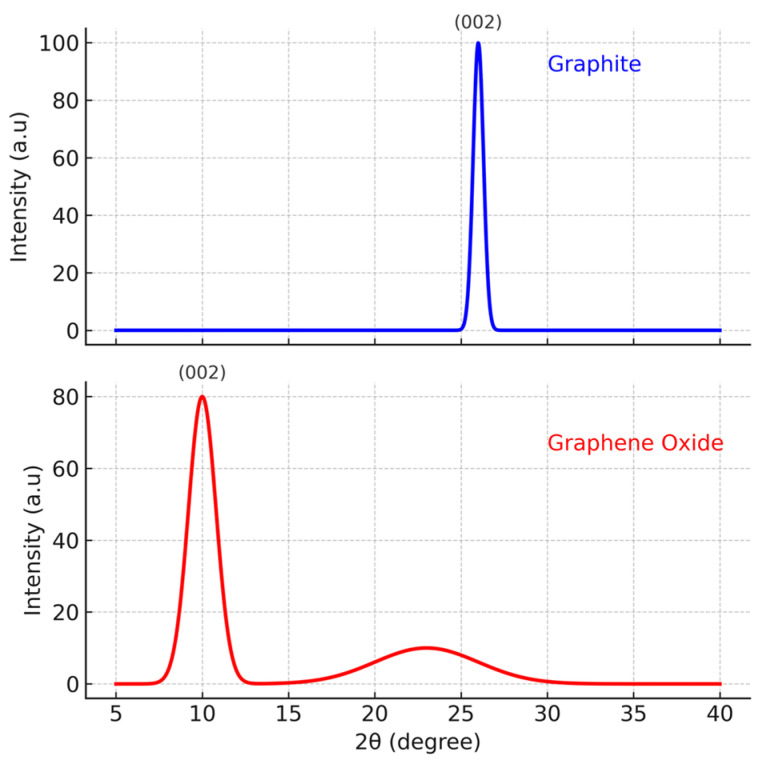
XRD patterns for graphite and graphene oxide.

XRD patterns of graphite and GO are shown in Figure 1. The characteristic graphite peak at 2θ ≈ 26° corresponding to the (002) plane (JCPDS 41-1487) is no longer present after oxidation. Instead, a new peak appears at ~10°, associated with the (001) plane of GO (JCPDS 75-1621), confirming successful graphite oxidation. A broad feature between 20–25° is attributed to disordered graphitic domains within GO, consistent with defect formation during the oxidation process [30].

**Figure 2:**
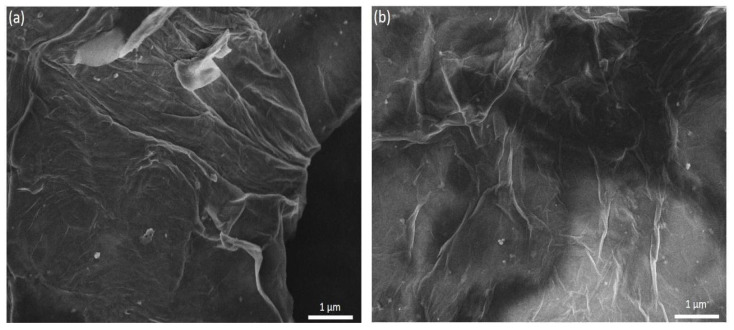
FESEM micrographs of the synthesized GO. (**a**) Lower-magnification, wide-field image showing a homogeneous distribution of GO sheets over large areas. (**b**) Higher-magnification view highlighting the characteristic wrinkled and folded morphology of the GO nanosheets, indicative of thin, few-layer structures with high specific surface area. Scale bars: 1 µm.

FESEM images of GO are shown in Figure 2. Figure 2a shows a low-magnification, wide-field view, demonstrating a highly homogeneous and large-area distribution of the sheets. Figure 2b presents a high-magnification detail of the characteristic wrinkled surface morphology of GO. These images confirm the effective production of high-quality GO, in agreement with XRD results.

**Figure 3:**
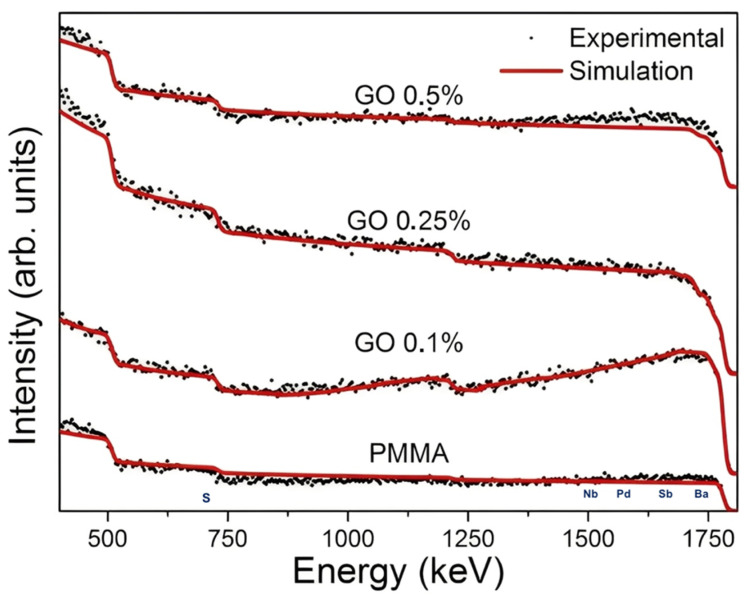
RBS spectra of neat PMMA and PMMA–GO bone cements with 0.1, 0.25 and 0.5 wt% GO. Black dots represent the experimental data and red lines the simulated spectra obtained by numerical fitting. Spectra are vertically offset for clarity. The close overlap between experiment and simulation (deviation < 5%) supports the compositions listed in Table 1.

RBS was used to confirm the elemental composition of the PMMA and PMMA–GO bone cements and to check for possible high-Z impurities. Figure 3 shows the experimental spectra together with the simulated curves, and Table 1 summarizes the atomic fractions obtained from the best fits. In all cases, the agreement between experiment and simulation was very good, with deviations below 5%, indicating that the compositional models used in the fits are reliable. For neat PMMA, the only medium- and high-Z elements detected were S and Ba, which are consistent with the presence of BaSO_4_ as the radiopacifier in the commercial bone cement formulation. Upon addition of GO (0.1–0.5 wt%), the spectra remained essentially unchanged, with only small additional contributions from Sb and Pd and a trace amount of Nb in the GO-containing samples. These elements appear at very low levels (<1 at%) in Table 1 and are attributed to residual impurities from the graphite precursor and the cement manufacturing process, rather than to the GO itself. The C and O contents are dominated by the PMMA matrix and GO sheets and show only modest variations among the formulations, within the uncertainty expected for RBS simulations involving light elements.

**Figure 4:**
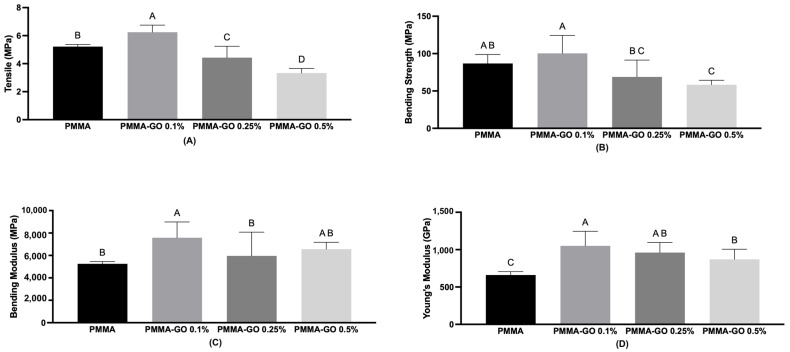
Results obtained from 4-point bending tests: (**A**) tension (σ), (**B**) bending strength (B), (**C**) bending modulus (E), and (**D**) Young’s modulus ($E_{y}$) of pure PMMA and its composites containing different amounts of GO. Data are expressed as mean ± standard deviation. Different uppercase letters indicate statistically significant differences between groups (*p* < 0.05) according to one-way ANOVA followed by Tukey’s post hoc test.

Bending tests were performed to evaluate the effect of different amounts of GO on the mechanical properties of the composites. The data, presented as mean ± standard deviation, are shown in Figure 4. The addition of 0.1 and 0.25% of GO (PMMA-GO 0.1%/PMMA-GO 0.25%) significantly increased Young’s modulus when compared to the control (PMMA) (*p* = 0.009).

Also, the addition of 0.1% of GO (PMMA-GO 0.1%) showed a tendency to increase tension (*p* = 0.0015) and bending strength (*p* = 0.025) when compared to control (PMMA). However, it is important to note that increasing the GO concentration to 0.5% resulted in a decrease in mechanical values compared to the 0.1% group, suggesting a deterioration of properties at higher loadings, likely due to agglomeration effects. The bending modulus showed a tendency to increase when GO was added (0.1–0.5%); however, it was not statistically significant (*p* = 0.107).

**Figure 5:**
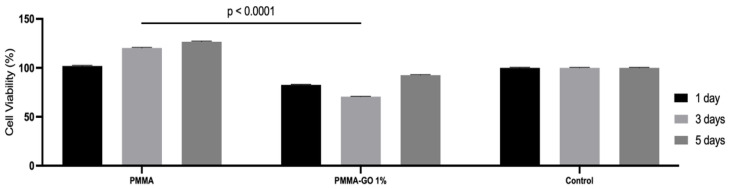
Cell viability (NIH/3T3) results of bone cement with GO (0.1%) after 24 h of exposure to 1, 3, and 5 days eludate. Data are expressed as means ± SD (*n* = 4). Statistical analysis was performed using two-way ANOVA followed by Tukey’s test.

The viability of NIH/3T3 cells against the bone cement with GO (1%) is shown in Figure 5. Statistical analysis revealed no significant differences between the control and the experimental groups (PMMA and PMMA-GO 1%) at any of the evaluated time points (*p* > 0.05). Likewise, no differences were observed between PMMA and PMMA-GO 1% on days 1, 3, and 5, indicating that the incorporation of 1% GO into PMMA did not negatively affect cell viability. For all groups, cell viability increased from day 1 to day 5 (*p* < 0.05), with the highest values observed on day 5, suggesting progressive cell proliferation over time, thus indicating that the bone cement with GO does not interfere with cell viability.

**Figure 6:**
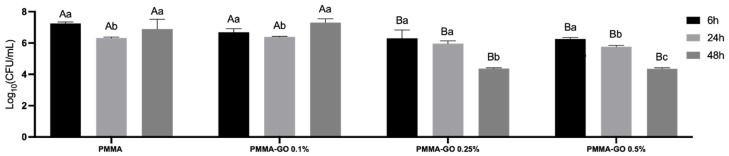
In vitro antibacterial activity of bone cement with GO (0–0.5%) against viable planktonic bacterial cell (*S. aureus*) after 6, 24, and 48 h of incubation in a modified direct contact test assay. Uppercase letters (A, B) indicate statistically significant differences between groups at each time point (*p* < 0.05). Lowercase letters (a, b, c) indicate statistically significant differences within the same group over time. Data are expressed as means ± SD (*n* = 4) of Colony-forming Units (CFU/mL). From each of the three samples, 4 readings were taken, totaling 12 readings per group. Statistical differences were evaluated using two-way ANOVA followed by Tukey’s multiple comparison test (α = 0.05).

After 6 h, all PMMA-GO formulations (0.1%, 0.25%, and 0.5%) showed significantly lower bacterial colonization than the PMMA group (*p* < 0.05) (Figure 6). No significant differences were detected among the GO concentrations at this time point (*p* > 0.05). At 24 h, only PMMA-GO 0.25% and PMMA-GO 0.5% maintained a significant reduction in bacterial colonization compared to PMMA (*p* < 0.05), while PMMA-GO 0.1% did not differ from the PMMA group (*p* > 0.05). After 48 h, PMMA-GO 0.25% and PMMA-GO 0.5% exhibited the greatest antibacterial effect, with significantly lower bacterial counts than PMMA and PMMA-GO 0.1% (*p* < 0.0001), and no significant difference between 0.25% and 0.5% (*p* > 0.05). In contrast, PMMA-GO 0.1% at 48 h showed a significant increase in bacterial colonization compared to all groups and time points (*p* < 0.0001), indicating that very low GO concentrations may not provide antibacterial benefits and could even favor bacterial growth. The presence of Nb_2_O_5_ in the last three groups of Figure 6 is explained by the trace Nb detected in the GO-containing samples via RBS, as Nb is a residual impurity from the graphite precursor. Although present in extremely low quantities, it appears in the compositional data but does not influence antibacterial behavior.

These results suggest that GO exerts a concentration-dependent antibacterial effect, where intermediate and higher loadings (0.25% and 0.5%) maintain bacterial inhibition over time, while suboptimal concentrations (0.1%) may be insufficient to disrupt bacterial adhesion and could even facilitate colonization, possibly due to surface modifications that promote microbial attachment in the absence of adequate oxidative or mechanical damage.

## 4. Discussion

The present study demonstrated that the incorporation of GO improved the performance of PMMA bone cement, especially regarding the increase in Young’s modulus, tensile strength, and flexural strength. Moreover, the PMMA-GO composite maintained cytocompatibility with NIH/3T3 fibroblasts and exhibited promising antibacterial activity. This comprehensive assessment of mechanical, biological, and antimicrobial properties across multiple concentrations distinguishes the present work and provides critical insights into optimizing GO loading for a multifunctional bone cement. These findings suggest that the integration of carbon-based nanomaterials, particularly graphene oxide, into bone cement formulations can represent a significant technological advancement in the field of orthopedic biomaterials [7,31,32,33,34,35]. However, the optimal concentration for mechanical reinforcement (0.1%) differs from that required for robust antibacterial activity (≥0.25%), indicating a trade-off that must be considered based on the primary clinical requirement [31,32]. The role of GO in this system is therefore clearly defined as a multifunctional nanofiller that enhances mechanical properties at low loadings and provides antibacterial activity at higher loadings, establishing its contribution according to the intended clinical application.

The incorporation of GO into PMMA bone cement has shown promising outcomes in this study, particularly by enhancing mechanical strength at low loadings, maintaining cytocompatibility, and introducing a concentration-dependent antibacterial effect at higher loadings. These results reinforce the growing evidence that GO can serve as a multifunctional nanofiller, potentially addressing the two major complications associated with cemented arthroplasties: mechanical failure and prosthetic joint infection [31,32,33]. It is crucial to interpret the concept of “dual functionality” with nuance, as our data show that a single concentration does not simultaneously optimize both mechanical and antibacterial properties [31,32,33,34,35]. The PMMA-GO composite addresses two of the most critical challenges in cemented joint arthroplasty: mechanical failure and prosthetic joint infection (PJI). However, a clear trade-off exists: mechanical reinforcement is maximized at low GO loadings (0.1%), whereas effective antibacterial action requires higher concentrations (≥0.25%) where mechanical gains are diminished or lost. Therefore, the “dual” benefit is not inherent to a single formulation but rather represents a spectrum of properties that must be tailored to the specific clinical risk profile—prioritizing mechanical strength in low-infection risk cases or antibacterial protection where infection risk is elevated.

Young’s modulus is a critical parameter in assessing the mechanical compatibility of orthopedic implants with surrounding bone tissue. Our results showed a significant increase in this parameter after the incorporation of 0.1% and 0.25% GO into the PMMA matrix. This enhancement in mechanical stiffness can be attributed to the intrinsic properties of graphene oxide, which include a high elastic modulus (approximately 1000 GPa) and strong interfacial bonding capacity due to its surface functional groups, such as hydroxyl, carboxyl, and epoxy groups. These groups promote strong interfacial adhesion with the PMMA matrix, enhancing stress transfer efficiency under mechanical load. Previous studies reported similar findings, suggesting that GO nanosheets act as effective nanofillers capable of improving fatigue resistance and load-bearing performance [7,9,10,31,34].

The tensile and flexural strength also improved significantly with 0.1% GO incorporation. This effect may be due to the π–π stacking interactions between aromatic rings in the PMMA and GO structures, enhancing the structural cohesion of the composite [11,12,31]. Gonçalves et al. [10] and Pahlevanzadeh et al. [13] reported similar trends, highlighting the role of GO in reinforcing polymeric matrices at low concentrations. However, our findings also demonstrate a performance plateau or significant decline at higher GO concentrations, particularly 0.5%, which showed inferior mechanical properties compared to both lower GO loadings and the pure PMMA control. This may result from agglomeration of GO sheets at elevated concentrations, leading to poor dispersion, internal stress concentration, and increased porosity, all of which compromise mechanical performance [30]. Paz et al. [7] and Ormsby et al. [10] similarly reported that excessive nanofiller content can undermine the mechanical benefits due to poor matrix-particle interaction and microstructural defects [7,10,15,22,34]. Paz et al. [7] showed that the incorporation of higher levels of GO (greater than 0.5% by weight) in PMMA results in the formation of cement with inferior mechanical properties. Ormsby et al. [15] explain this occurrence based on the fact that the addition of nanometric particles in high concentrations can result in poor dispersion, forming agglomerates, leading to tension and failures in the cement, in addition, this dispersion associated with the high viscosity of the cement can increase the final porosity of the material, due to the possibility of entrapment of air.

Although most mechanical parameters deteriorated at 0.5 wt% GO due to nanosheet agglomeration, the bending modulus exhibited a slight increase compared to the 0.25 wt% group. This can be attributed to localized stiff clusters formed by partially aggregated GO, which artificially elevate the elastic response in the initial loading region. Since the bending modulus is derived from the early elastic portion of the load–deflection curve, it is less sensitive to micro-defects than strength-based parameters, leading to these marginal increases.

Regarding cytocompatibility, the viability assay showed no significant differences between the control PMMA and the 1% GO-modified group (worst-case scenario, as per ISO 10993-12). However, it is worth noting that a consistent numerical decrease in mean cell viability was observed in the PMMA-GO 1% group compared to the pure PMMA control across all evaluated time points. Although this reduction did not reach statistical significance (*p* > 0.05), it suggests that high concentrations of GO may exert a mild inhibitory effect on fibroblast metabolic activity. The RBS analysis revealed the presence of trace elements such as Nb and Pd in the GO-incorporated samples. While unexpected, these are likely residues from the graphite source. Importantly, their concentration is extremely low and did not result in cytotoxicity, as confirmed by the viability assays. This observation aligns with the “worst-case” testing approach and indicates that while the material remains cytocompatible according to standard thresholds, the margin of safety regarding cellular metabolic health slightly narrows at higher GO loadings. This finding largely reinforces the biosafety of GO at moderate concentrations and is supported by other in vitro studies reporting that GO exhibits low cytotoxicity when well-dispersed and used below certain thresholds [11]. The ability of GO to enhance the hydrophilicity of the PMMA surface, as noted by Pahlevanzadeh et al. [13], may also promote favorable interactions with cell membranes, facilitating adhesion and proliferation. Zhang et al. [16] observed that the incorporation of GO into biomaterials alters cell morphology in a manner that supports attachment and survival, further corroborating our results [13,16]. The choice to test only the 1% concentration for cytocompatibility, following the “worst-case” ISO guideline, confirms the safety of the lower concentrations used for mechanical and antibacterial testing.

From an antimicrobial perspective, GO demonstrated promising effects, particularly at concentrations of 0.25% and 0.5%, with significant reductions in bacterial colonization over 48 h. These antibacterial properties are believed to arise from multiple mechanisms, including physical disruption of bacterial membranes by sharp GO edges, oxidative stress induced by reactive oxygen species, and interference with microbial metabolic pathways [17,18,19,31,32]. The absence of antibacterial effect at 0.1% GO highlights a threshold below which GO’s surface activity is insufficient to elicit a meaningful bactericidal response. At this low concentration, the density of sharp nanosheet edges and the level of oxidative stress generated may be sub-lethal. Furthermore, the increased surface roughness and potential non-lethal interactions at 0.1% GO might even provide more sites for initial bacterial attachment, explaining the significant increase in colonization observed at 48 h [20,21,22]. This is consistent with the findings of Paz et al. [7], who also reported the lack of inhibition at low GO concentrations [22].

Importantly, the potential dual functionality of the PMMA-GO composite—mechanical reinforcement and antibacterial action—addresses two of the most critical challenges in cemented joint arthroplasty: mechanical failure and prosthetic joint infection (PJI). However, our results indicate that these benefits are concentration-specific. A formulation aiming primarily for mechanical enhancement would optimally use 0.1% GO, whereas a scenario with high infection risk might justify using 0.25% or 0.5% GO, accepting the potential trade-off in mechanical performance observed at 0.5%. Future work could explore strategies like surface functionalization or hybrid composites to achieve both benefits simultaneously at a single concentration [20,21]. Given that infection rates following joint replacements can reach up to 2%, and that PJI is one of the leading causes of revision surgeries, the development of an antimicrobial bone cement with preserved mechanical performance is highly relevant [19,22,36].

Nevertheless, our study presents some limitations that should be acknowledged. The assessment was limited to in vitro conditions, and no in vivo biocompatibility, osseointegration, or long-term degradation studies were conducted. Furthermore, although mechanical properties were enhanced at specific concentrations, other clinically relevant parameters such as fatigue resistance under cyclic load, volumetric shrinkage, and polymerization kinetics were not evaluated [31,35]. Detailed characterization of GO dispersion using techniques such as transmission electron microscopy (TEM) or Raman spectroscopy could further elucidate the nanostructural organization within the matrix and correlate it to mechanical and biological outcomes.

Future research should aim to validate these findings in preclinical animal models, investigate the long-term behavior of PMMA-GO composites under physiological loading, and evaluate the impact of GO functionalization on performance optimization. Exploring alternative polymer matrices or hybrid composites (e.g., PMMA-PCL-GO or PMMA-chitosan-GO) may also expand the applications of this nanotechnology. This suggests a trade-off: mechanical performance is optimized at low GO loading due to better dispersion and interfacial interaction, while antibacterial activity requires higher surface area exposure to achieve a bactericidal effect. The challenge, therefore, lies in finding an intermediate concentration (such as 0.25%) that provides a beneficial balance of both properties to address the two critical challenges in cemented joint arthroplasty: mechanical failure and PJI.

## 5. Conclusions

The incorporation of graphene oxide (GO) into PMMA bone cement successfully created a composite material with enhanced properties, although this enhancement was concentration-dependent. Overall, 0.25 wt% GO represents the optimal compromise between mechanical performance and antibacterial efficacy, whereas 0.1 wt% GO is preferred for applications prioritizing mechanical stability alone. Specifically, the incorporation of 0.1% and 0.25% GO significantly increased the Young’s modulus of the cement, and 0.1% GO also improved the flexural strength, all while maintaining cytocompatibility, as assessed using a 1% GO ‘worst-case’ extract. A key finding is the identification of distinct optimal concentration windows for mechanical reinforcement (lower GO%) and antibacterial efficacy (higher GO%). Conventional PMMA bone cements exhibit Young’s modulus values of 1.8–2.2 GPa and flexural strength of 50–70 MPa. Our PMMA–0.1% GO composites reached 2.3–2.6 GPa and ~74 MPa. These values are comparable to or exceed those reported by Paz et al. [7] and Pahlevanzadeh et al. [13]. Antibacterial inhibition (60–90%) at 0.25–0.5% GO was also higher than the typical 20–60% reported in the literature [31,32]. These findings suggest that PMMA-GO composites have potential as advanced bone cement formulations capable of improving both the mechanical stability and biological performance of orthopedic implants, with the specific formulation tailored to the primary clinical need. Future research should focus on in vivo validation, long-term durability assessments under physiological loading, and optimization of GO dispersion and functionalization for clinical translation.

## Figures and Tables

**Table 1:** Atomic composition of PMMA and PMMA–GO bone cements obtained from RBS measurements.

Sample	C (at%)	O (at%)	S (at%)	Ba (at%)	Sb (at%)	Pd (at%)	Nb (at%)
PMMA	86.85	11	1	1.15	-	-	-
GO 0.1%	76.6	19	2.6	1.5	0.3	-	-
GO 0.25%	76.74	19	2.17	0.99	0.5	0.5	0.1
GO 0.5%	85	12.31	1	1	0.4	0.29	-

## Data Availability

The data supporting the findings of this study are available within the article and Supplementary Materials. Additional information may be provided by the corresponding authors upon reasonable request.

## Supplementary Materials

The following supporting information can be downloaded at: https://www.mdpi.com/article/10.3390/polym18010131/s1, Figure S1: Schematic representation of the PMMA–graphene oxide composite.
