# Supplementary figures and images for: Novel Acrylic Bone Cement Containing Graphene Oxide: Synthesis and Characterization
Source: Polymers (Basel). 2025 Dec 31;18(1):131. doi: 10.3390/polym18010131 (PMC12787345; doi:10.3390/polym18010131)

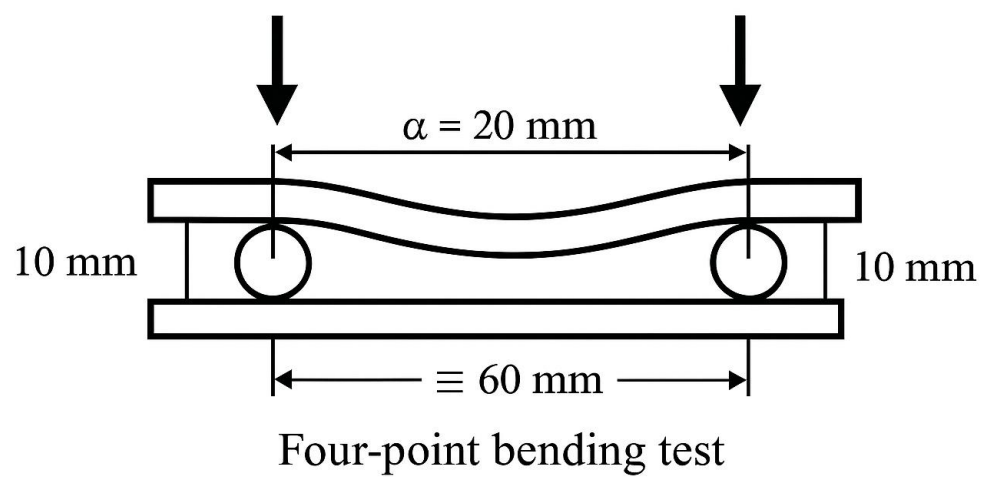

**Figure S1.** Schematic representation of the PMMA–graphene oxide composite.

Supplement: Supplementary file 1 [file polymers-18-00131-s001.zip › polymers-4009212-supplementary.pdf]
